# Supplementary material for: Disorder-specific alterations of tactile sensitivity in neurodevelopmental disorders
Source: Commun Biol. 2021 Jan 22;4:97. doi: 10.1038/s42003-020-01592-y (PMC7822903; doi:10.1038/s42003-020-01592-y)
Supplement: Supplementary file 2 — Supplementary Information [file 42003_2020_1592_MOESM2_ESM.pdf]

Disorder-specific alterations of tactile sensitivity in neurodevelopmental disorders – Supplementary Materials

Jason L. He[1,2,3], Ericka Wodka[4,5], Mark Tommerdahl[6], Richard A. E. Edden[1,2], Mark Mikkelsen[1,2], Stewart H. Mostofsky[4,5,7,8], & Nicolaas A. J. Puts[1,2,3]

[1]*Russell H. Morgan Department of Radiology and Radiological Science, The Johns Hopkins University School of Medicine, Baltimore, 21287, Maryland, USA*

[2]*F. M. Kirby Research Center for Functional Brain Imaging, Kennedy Krieger Institute, Baltimore, 21287, MD, USA.*

[3] *Department of Forensic and Neurodevelopmental Sciences, Sackler Institute for Translational Neurodevelopment, Institute of Psychiatry, Psychology, and Neuroscience, King's College London, SE5 8AB, London, UK*

[4] *Center for Autism and Related Disorders, Kennedy Krieger Institute, Baltimore, MD, USA*

[5] *Department of Psychiatry and Behavioral Sciences, The Johns Hopkins University School of Medicine, Baltimore, 21287, MD, USA*

[6] *Department of Biomedical Engineering, University of North Carolina at Chapel Hill, Chapel Hill, 27514, NC, USA*

[7] *Center for Neurodevelopmental and Imaging Research, Kennedy Krieger Institute, Baltimore, 21287, MD, USA*

[8] *Department of Neurology, The Johns Hopkins University School of Medicine, Baltimore, 21287, MD, USA*

Supplementary Methods

Simple and choice reaction to tactile stimuli. Reaction time was tested under two protocols, a simple reaction time condition and a choice reaction time protocol. In the simple reaction time protocol, a suprathreshold stimulus (frequency = 25 Hz; amplitude = 300 μm; duration = 40 ms) was pseudo-randomly delivered to either left digit 2 or left digit 3, and participants were asked to respond with their right hand as soon as they felt the stimulus. For the simple reaction time protocol, participants simply needed to click any mouse button, whereas in the choice reaction time protocol participants additionally had to indicate, using the left (right digit 2) and right mouse (right digit 3) buttons, on which finger they felt the stimulus. For both protocols, there were 20 trials delivered with an intertrial interval of 3 s.

To maintain consistency with estimation of reaction times in the conditions below, the median reaction time for each protocol was also estimated (simple median reaction time and choice median reaction time). Reaction time variability across all trials was estimated by taking the standard deviation across all trials (regardless of accuracy in the choice reaction time condition). Accuracy for the choice reaction time was estimated as the total number of trials in which the participant was able to accurately identify the site of stimulation (left digit 2 or left digit 3).

Dynamic detection. In the dynamic detection condition, a 25-Hz stimulus increased from zero amplitude (rate of amplitude increase = 2 μm/s). Participants were asked to respond on which finger they felt the stimulus as soon as they felt it. Each trial began with a variable delay (0-2500 ms) and trials were delivered with an inter-trial interval of 10 s. There were 7 trials in the dynamic detection protocol. Dynamic detection thresholds were calculated as the mean stimulus amplitude at the time of pressing the button, across all correct trials.

Amplitude discrimination with adaptation. Amplitude discrimination was also tested with adaptation[1]. In the adaptation conditions, adaptive stimuli were delivered to the digit receiving the higher amplitude (referred to as single-site adaptation) or to both digits (referred to dual-site adaptation). Participants were asked to choose which of two simultaneously delivered stimuli had the higher amplitude (frequency = 25 Hz; duration = 500 ms; standard stimulus amplitude = 100 μm; initial comparison stimulus amplitude = 200 μm).

In the single-site adaptation protocol, each trial was preceded by a single-site adaptive stimulus (frequency = 25 Hz; duration = 1 s, amplitude = 100 μm) and in the dual-site adaptation protocol, each trial was preceded by a dual-site adaptive stimuli (frequency = 25 Hz; duration = 1 s, amplitude = 100 μm). Participants were told to ignore the adapting stimulus. For all amplitude discrimination conditions, a one-up–one-down tracking paradigm (comparison stimulus amplitude was decreased for a correct answer and increased for a wrong answer) was used for the first 10 trials and a two-up–one-down was used for the remainder of the task. All conditions contained 20 trials delivered with a 5s inter-trial interval. Amplitude discrimination thresholds without adaptation, with single-site adaptation and dual-site adaptation were calculated as the mean of the amplitudes of the last five trials.

Sequential frequency discrimination. Frequency discrimination of sequentially delivered stimuli was tested. Stimuli (duration = 500 ms; amplitude = 200 μm) were delivered to left digit 2 and left digit 3 simultaneously to both digits, separated by a 500ms inter-trial interval (ISI). One finger always received the standard stimulus (frequency = 30 Hz) while the other received the comparison stimulus (initial frequency = 40 Hz). The two stimuli were delivered to either digit pseudo-randomly. Participants were asked which finger received the higher frequency stimulus.

A one-up–one-down tracking paradigm (the comparison stimulus frequency was decreased for a correct answer and increased for a wrong answer) was used for the first 10 trials. A two-up–one-down was used for the remainder of the trials. All conditions contained 20 trials and were delivered with an inter-trial interval of 5 s. Frequency discrimination thresholds were obtained as the mean of the frequencies of the last five trials.

Temporal order judgement with carrier. When a carrier stimulus is delivered shortly prior to sequentially delivered stimuli to these locations, an individual's ability to make order judgements is temporarily impaired. Two single-cycle vibrotactile pulses (duration = 40 ms; frequency = 25 Hz; amplitude = 200 μm) were delivered to left digit 2 and left digit 3 separated temporally by a starting ISI of 150 ms (the first pulse was assigned to either digit pseudo-randomly) within a 1-s interval. A 25-Hz, 20-μm concurrent carrier stimulus was delivered throughout each 1-s trial interval.

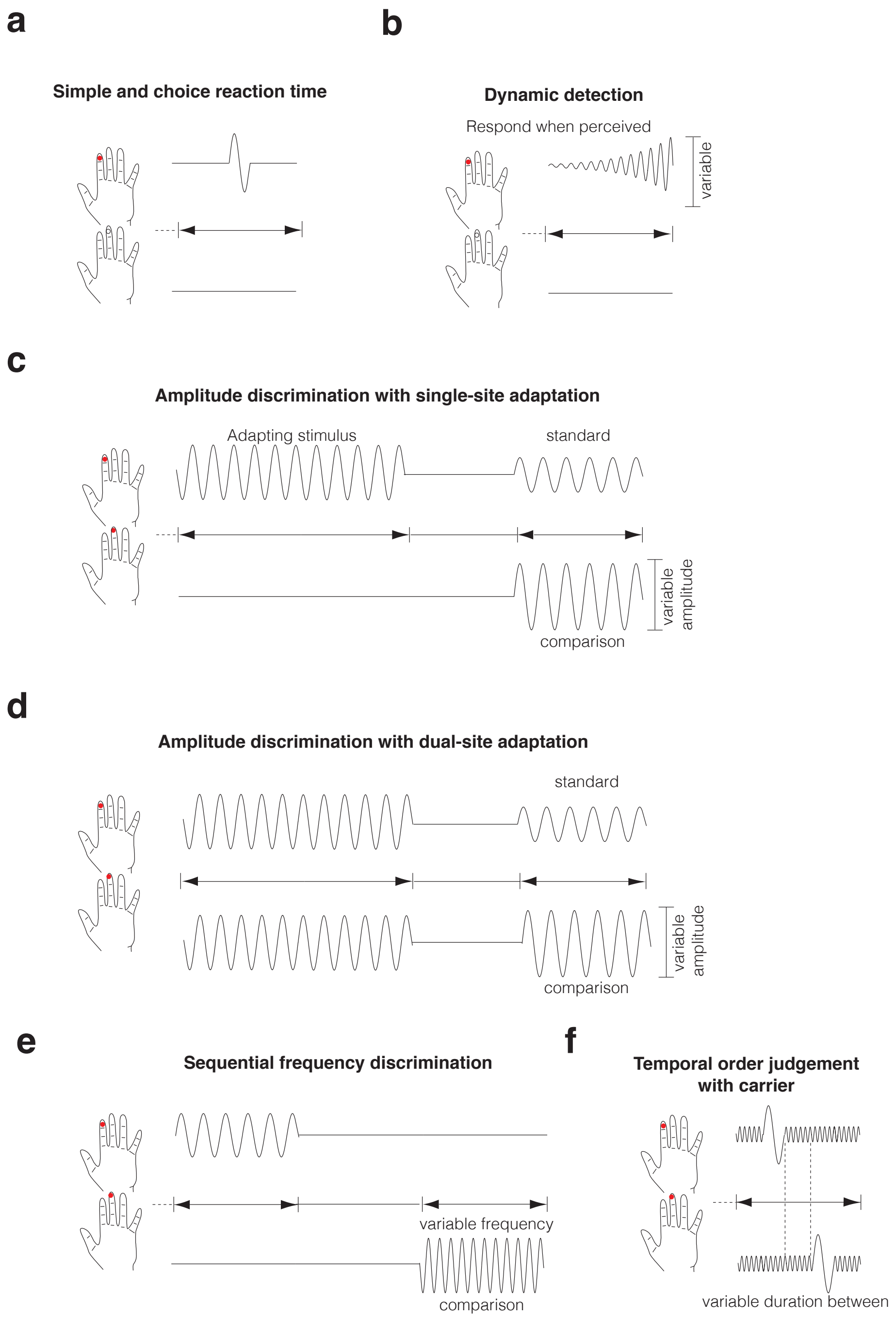

Supplementary Fig. 1. Visual schematic of protocols that were completed but were not included in the main body of the manuscript. (**a**) Simple and choice reaction time, (**b**) dynamic detection, (**c**) amplitude Discrimination with single-site adaptation, (**d**) amplitude discrimination with dual-site adaptation, (**e**) sequential frequency discrimination, (**f**) temporal order judgement with carrier stimulus.

Supplementary Results

Simple reaction time. When comparing performance on the simple reaction time protocol between typically developing control, ASD, ASD + ADHD and ADHD groups, there was a main effect of group on median reaction times ($F(3, 409) = 3.46$, $p = 0.016$; $\eta^2_p = 0.02$; $BF_{10} = 10132.02$) and reaction time variability ($F(3, 336) = 6.99$, $p = 0.001$; $\eta^2_p = 0.01$; $BF_{10} = 84.45$). Posthoc comparisons found that the ADHD ($t(409) = 2.41$, $p_{Tukey} = 0.08$; $d = 0.60$; $BF_{10} = 2.48$) and ASD + ADHD ($t(409) = 2.78$, $p_{Tukey} = 0.029$; $d = 0.70$; $BF_{10} = 6.62$) groups had slower median reaction times than typically developing controls, but children with ASD ($t(409) = 0.68$, $p_{Tukey} = 0.905$) did not. The ADHD group also had greater variability in reaction times than controls ($t(336) = 4.56$, $p_{Tukey} < 0.001$; $d = 0.60$; $BF_{10} = 1354.62$), while the ASD ($t(336) = 0.45$, $p_{Tukey} = 0.970$; $d = 0.34$; $BF_{10} = 0.26$) and ASD + ADHD ($t(336) = 0.1.06$, $p_{Tukey} = 0.72$; $d = 0.70$; $BF_{10} = 0.29$) groups did not. Interestingly, the ADHD group was more variable than the ASD + ADHD group ($t(336) = 2.96$, $p_{Tukey} = 0.017$; $d = 0.08$; $BF_{10} = 3.91$). See Supplementary Fig. 2(a).

Choice reaction time. Next we compared performance on the Choice Reaction Time protocol between groups**.** As with the simple reaction time protocol**,** there was also a main effect of group on median ($F(3, 399) = 6.57$, $p < 0.001$; $\eta^2_p = 0.04$; $BF_{10} = 2.12^{13}$) and standard deviation ($F(3, 321) = 7.38$, $p < 001$; $\eta^2_p = 0.06$; $BF_{10} = 8730.78$) of reaction times. There was also a trend towards a significant main effect of group on accuracy ($F(3,374) = 2.34$, $p = 0.073$; $\eta^2_p = 0.02$; $BF_{10} = 0.23$). Posthoc comparisons found that those in the ADHD ($t(399) = 3.42$, $p_{Tukey} < 0.001$; $d = 0.60$ $BF_{10} = 75.57$) and ASD + ADHD ($t(399) = 3.76$, $p_{Tukey} < 0.001$; $d = 0.70$; $BF_{10} = 61.21$) group had significantly longer median reaction times on the choice reaction time protocol than the typically developing control group, but those in the ASD ($t(399) = 1.26$,

$p_{\text{Tukey}} = 0.59$; $d = 0.34$; $BF_{10} = 0.32$) group did not. The ADHD ($t(321) = 3.54$, $p_{\text{Tukey}} = 0.003$; $d = 0.60$;$BF_{10} = 94.17$) and ASD + ADHD ($t(321) = 3.39$, $p_{\text{Tukey}} < 0.004$; $d = 0.70$; $BF_{10} = 115.14$) groups also had significantly more variable reaction times on the choice reaction time task than the typically developing control group, while the ASD group did not ($t(321) = 0.77$, $p_{\text{Tukey}} = 0.86$; $d = 0.34$; $BF_{10} = 0.43$). The ASD + ADHD group was also more variable than the ASD only ($t(321) = 2.76$, $p_{\text{Tukey}} = 0.030$; $d = 0.33$; $BF_{10} = 3.82$) group, potentially suggesting an 'additive' effect of having comorbid ADHD. In a similar vein, only the ASD + ADHD group were less accurate than the typically developing control group in the choice reaction time task ($t(374) = 2.63$, $p_{\text{Tukey}} = 0.043$; $d = 0.70$; $BF_{10} = 4.51$), with all other groups showing similar levels of accuracy to the typically developing control group (all $p > .838$). See Fig. 2(b).

Cost of choice on reaction times. Following group comparisons on the simple reaction time and choice reaction time protocols, we assessed whether the 'cost' of the choice component affected median reaction times and whether this affect was comparable between groups. There was a significant main effect of condition ($\chi 2$ (1) = 803.19, $p < 0.001$) on median reaction times for all groups. On average, children were slower in the choice reaction time compared to simple reaction time protocol by 425.80 ms ± 12.11 ms, $t(365.298) = 35.18$, $p < 0.001$. There was a trend towards a protocol by group interaction, ($\chi 2$ (3) = 6.43, $p = 0.093$). Inspection of the change in slopes found that the increase in median reaction times was significantly greater for the ADHD ($t(370.59) = 2.27$, $p = 0.0183$) and ASD + ADHD ($t(370.59) = 2.02$, $p = 0.020$) groups than the typically developing control group by 72.06 ms ± 30.40 and 58.12 ± 31.26 ms respectively. The increase in median reaction times from the simple reaction time to choice reaction time condition (i.e., the 'cost' of choice) was otherwise comparable between the ASD and typically developing control groups ($t(384.75) = 0.24$, $p = 0.996$). See Fig. 2(c).

Longer and more variable reaction times in the reaction time protocols were more common for those in the ADHD and/or ASD + ADHD groups, but appeared to be comparable to typically developing controls for those in the ASD group. Similarly, the cost of choice on reaction times appeared to have a greater effect for those in the ADHD and ASD + ADHD groups than controls, while those in the ASD group had a comparable change in reaction times to those in the control group. Taken together, these results suggest that slower and more variable reaction times, as well as the cost of choice on reaction times, are associated with the (co-) diagnosis of ADHD (i.e., is specific to the symptoms of ADHD, rather than the symptoms of ASD).

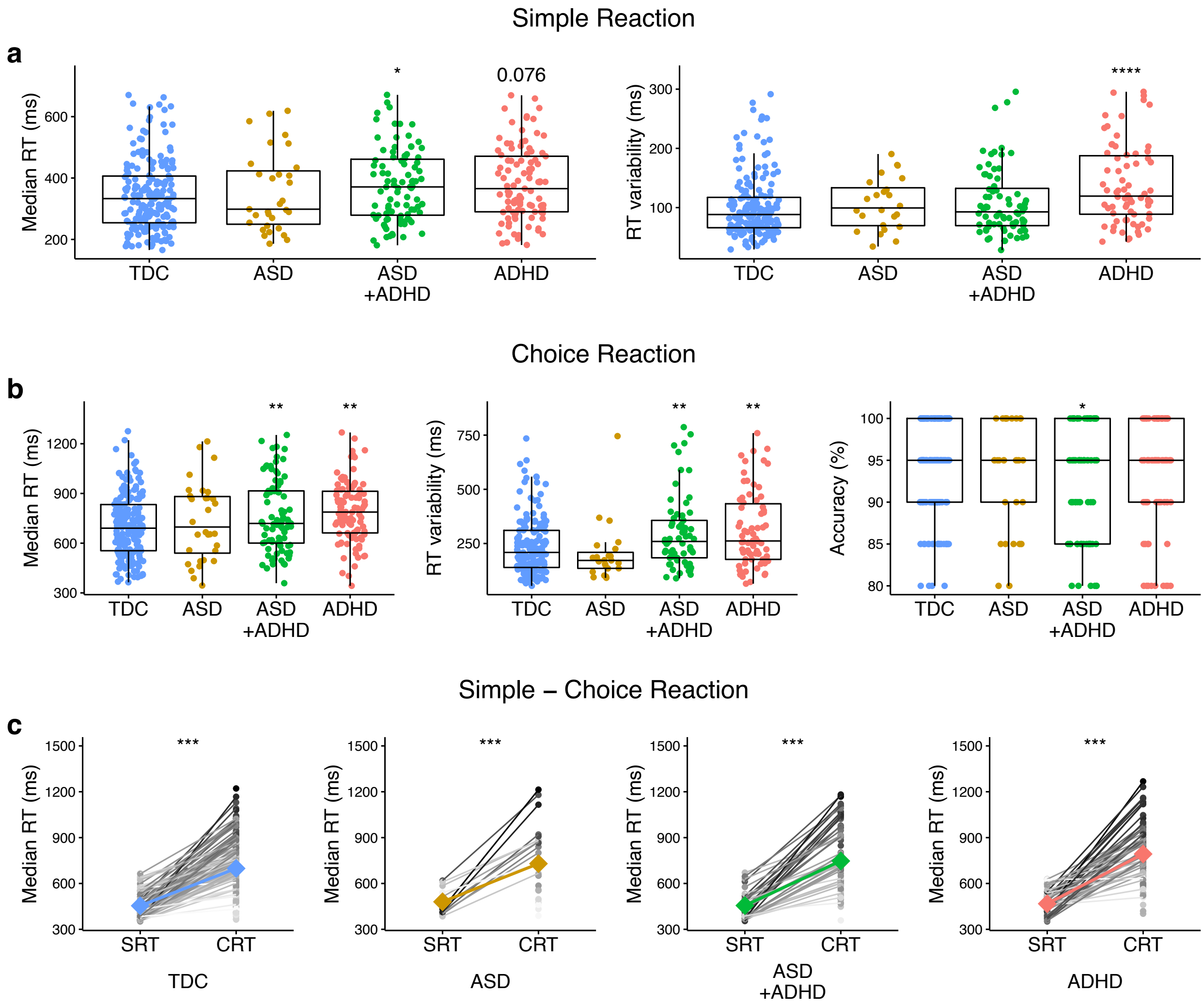

Supplementary Fig. 2. (a). Individual data points with overlaid boxplots visualizing the group comparisons on key performance outcomes on the simple reaction time and (**b**)**.** choice reaction time protocols **(c).** Individual data points and connecting slopes for median reaction times on the simple reaction and choice reaction time protocols for controls, the ASD, ASD + ADHD and ADHD groups. There is a clear and consistent increase in median reaction times when the choice component is added to the protocol, with participants having greater median reaction times (i.e., being slower) on the choice reaction time than simple reaction time protocol. Note that all statistical analyses presented in the main text of the manuscript for the plots presented in this figure are age corrected. Values in the plots presented within this figure were not corrected for age due to the loss of interpretability of units of measurement that occurs when presenting residual rather than raw values. The precise number of participants in each group and further descriptive statistics can be found in Supplementary Methods - Supplementary Table 1. Also note that although significance is indicated for the change in reaction times within each group, these analyses were

not conducted as part of the planned analyses and are simply presented to help readers interpret the results. * = $p < .05$, ** = $p < .01$, *** = $p < .001$, TDC = typically developing controls, ASD = autism spectrum disorders, ADHD = attention-deficit hyperactivity disorder, RT = reaction times, SRT = simple reaction time, CRT = choice reaction time

Dynamic detection. Unlike the static detection protocol, there was no significant main effect of group on thresholds ($F(3, 384) = 1.19$, $p = 0.313$; $\eta^2_p = 0.01$; $BF_{10} = 0.01$). There was also no main effect of group on median reaction times ($F(3, 392) = 1.51$, p = 0.212; $\eta^2_p = 0.01$; $BF_{10} = 0.01$). While there was a main effect of group on accuracy ($F(3, 399) = 3.06$, p = 0.028; $\eta^2_p = 0.02$; $BF_{10} = 4.53$) on dynamic detection thresholds, posthoc comparisons could not identify any significant or meaningful group differences. See Supplementary Fig. 3(b).

Change in detection thresholds. When collapsing across groups, there was a main effect of condition ($\chi 2$ (1) = 63.37, $p < .001$) on thresholds, with participant's detection thresholds being 2.42 micron ± .29 greater in the dynamic detection than the static detection protocols. However, there was also a group by condition effect, ($\chi 2$ (3) = 14.99, p = 0.002). Here, while all groups showed an increase in detection thresholds from the static detection to the dynamic detection protocol (typically developing controls: 2.41 ± 2.91; ASD: 1.34 ± .40; ADHD: 1.46 ± .39 and ASD+ADHD: 1.66 ± .52), the increase was significantly reduced in the ASD (-0.53, $t(359.82) = -0.70$, $p < 0.001$) and ADHD (-1.93 ± .51, $t(370.56) = -3.76$, $p < .001$) and ASD + ADHD (-1.0 ± .53, $t(370.278) = -1.85$, $p < .001$) groups compared to the typically developing control group. The increase in detection thresholds was otherwise comparable between the other pairwise combinations. See Supplementary Fig. 3(c).

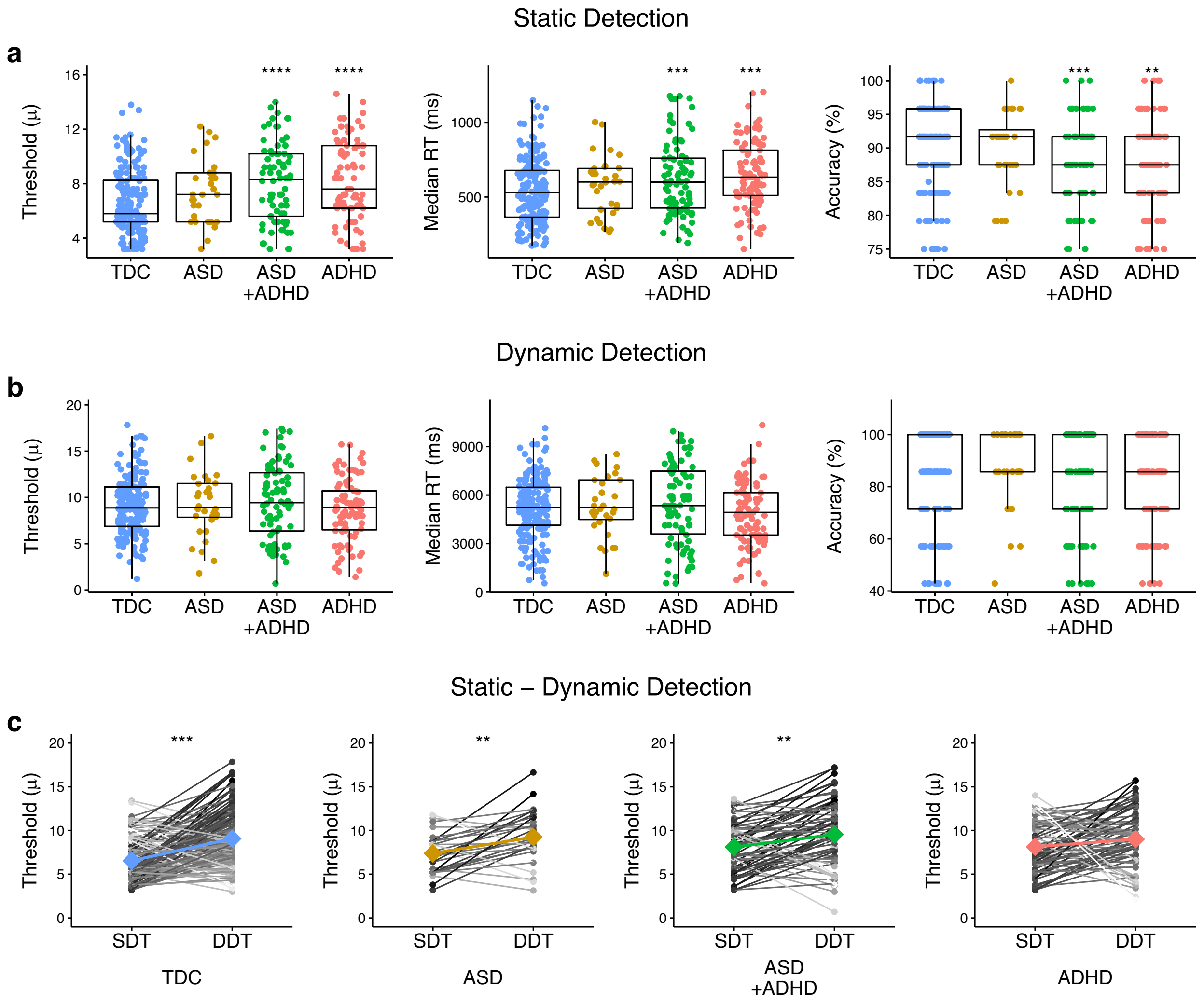

Supplementary Fig. 3. (a) Individual data points with overlaid boxplots visualizing the group comparisons on key performance outcomes on the static detection and **(b)** dynamic detection protocols. **(c)** Individual data points and connecting slopes for detection thresholds on the static detection and dynamic detection protocols for the typically developing control, ASD, ASD + ADHD and ADHD groups. At the group level, all groups (typically developing control, ASD, ASD + ADHD and ADHD) showed an increase in detection thresholds from the static detection to dynamic detection protocol. Note that all statistical analyses presented in the main text of the manuscript for the plots presented in this figure are age corrected. Values in the plots presented within this figure are not corrected for age due to the loss of interpretability of units of measurement that occurs when presenting residual rather than raw values. The precise number of participants in each group and further descriptive statistics can be found in Supplementary Methods - Supplementary Table 1. Also note that although significance is indicated for the change in thresholds within each group, these analyses were not conducted as part of the planned analyses and are simply

presented to help readers interpret the results. Table 1. * = $p < .05$, ** = $p < .01$, *** = $p < .001$, TDC = typically developing controls, ASD = autism spectrum disorders, ADHD = attention-deficit hyperactivity disorder, RT = reaction times, SDT = static detection thresholds, DDT = dynamic detection thresholds

Sequential frequency discrimination. Results from group comparisons on the performance outcomes of the sequential frequency discrimination protocol are shown in Supplementary Fig. 4. There was no significant main effect of group on sequential frequency discrimination thresholds ($F(3, 386) = 2.00$, $p = 0.11$; $\eta^2_p = 0.02$, $BF_{10} = .41$) and the main effect of group on accuracy was just shy of conventional significance ($F(3, 389) = 2.67$ $p = 0.050$; $\eta^2_p = 0.02$, $BF_{10} = .63$). There was a non-significant trend towards a main effect of group on median reaction time ($F(3, 373) = 2.20$, $p = 0.089$; $\eta^2_p = 0.02$, $BF_{10} = 35.83$). There was no significant main effect of group on the number of reversals ($F(3, 355) = 0.35$, $p = 0.791$; $\eta^2_p = 0.003$, $BF_{10} = .003$). Compared to the typically developing control group, frequency discrimination thresholds were worse in the ASD + ADHD ($t(386) = 2.44$, $p_{\text{Tukey}} = 0.070$; $d = 0.70$; $BF_{10} = 2.54$) group. The ASD ($t(386) = 0.53$, $p_{\text{Tukey}} = 0.951$; $d = 0.34$; $BF_{10} = 0.0.23$) and ADHD ($t(386) = 0.81$, $p_{\text{Tukey}} = 0.846$; $d = 0.34$; $BF_{10} = 0.0.23$) groups otherwise had similar frequency discrimination thresholds to those in the typically developing control group. Only the ASD + ADHD group were less accurate than the typically developing control group ($t(389) = 2.59$, $p_{\text{Tukey}} = 0.048$; $d = 0.70$; $BF_{10} = 4.20$).

Change in frequency discrimination thresholds. When collapsing across groups, there was a main effect of Condition ($\chi 2$ (1) = 33.36, $p < .001$) on thresholds, with participant's detection thresholds being 1.39 Hz ± .24 greater when delivered simultaneously rather than the sequentially. There was no Group by Condition effect, ($\chi 2$ (3) = 0.89, p = 0.827). See Supplementary Fig. 4(c).

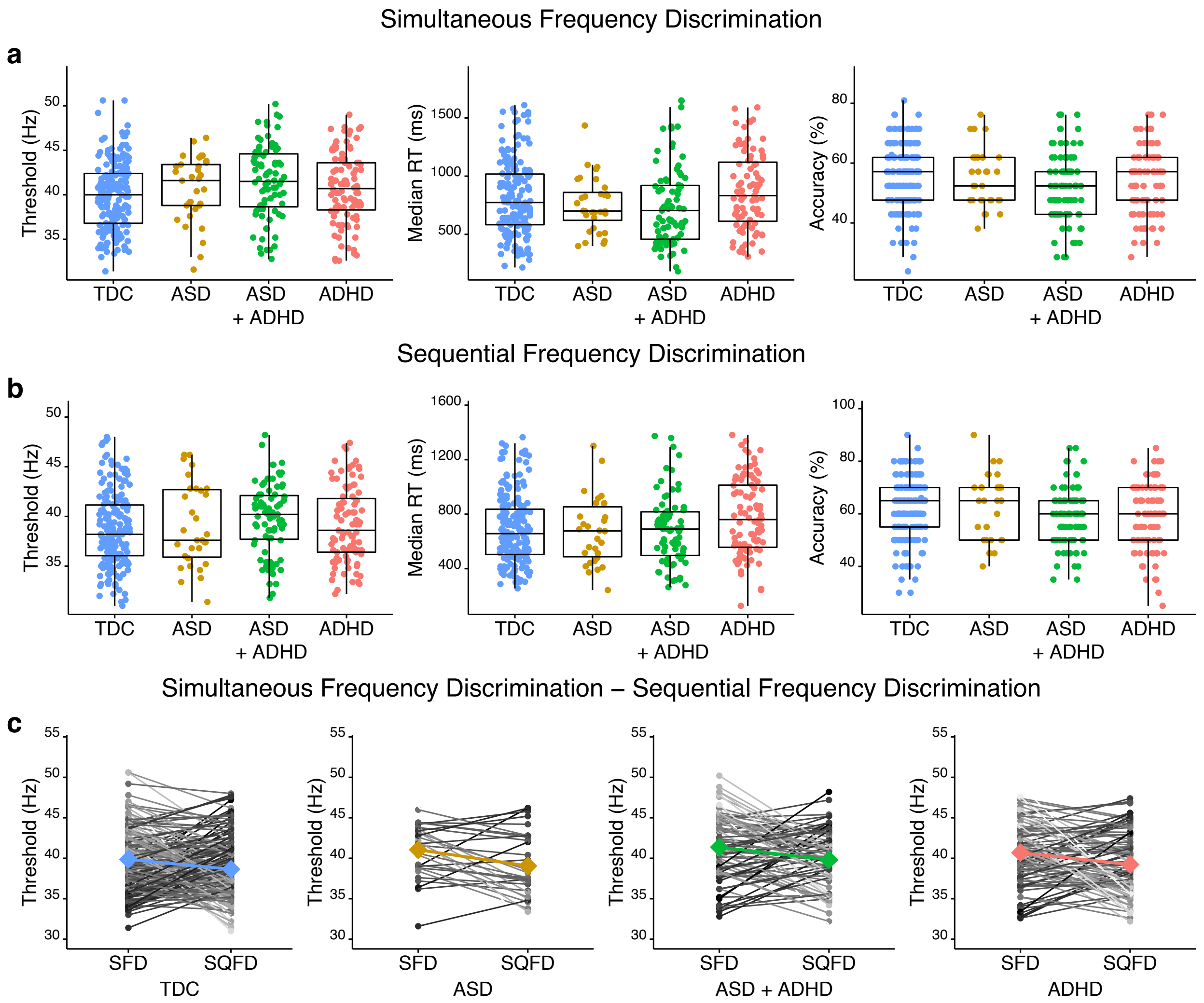

Supplementary Fig. 4 (a) Individual data points with overlaid boxplots visualizing the group comparisons on key performance outcomes on the sequential and (**b)** Simultaneous Frequency Discrimination protocols. **(c)** Individual data points and connecting slopes for detection thresholds on the simultaneous and Sequential Frequency Discrimination protocols for the typically developing control, ASD, ASD + ADHD and ADHD groups. Note that all statistical analyses presented in the main text of the manuscript for the plots presented in this figure are age corrected. Values in the plots presented within this figure are not corrected for age due to the loss of interpretability of units of measurement that occurs when presenting residal rather than raw values. Also note that although significance is indicated for the change in thresholds within each group, these analyses were not conducted as part of the planned analyses and are simply presented to help readers interpret the results. TDC = typically developing

controls, ASD = autism spectrum disorders, ADHD = attention-deficit hyperactivity disorder, RT = reaction times, SFD = simultaneous frequency discrimination, SQFD = sequential frequency discrimination

Amplitude discrimination with single-site and dual-site adaptation. With single-site adaptation, there was a significant main effect of group on amplitude discrimination thresholds ($F(3, 401) = 3.04$, $p = 0.029$; $\eta^2_p = 0.02$, $BF_{10} = .23$) and accuracy ($F(3, 401) = 3.22$, $p = 0.022$; $\eta^2_p = 0.02$, $BF_{10} = .32$), but no main effect of group on median reaction times ($F(3, 385) = 1.80$, $p = 0.112$ $\eta^2_p = 0.02$, $BF_{10} = .06$) or the number of reversals ($F(3, 403) = 0.78$, $p = 0.503$; $\eta^2_p = 0.01$, $BF_{10} = .004$). As with amplitude discrimination thresholds without adaptation, posthoc comparison on performance on the amplitude discrimination with single-site adaptation found that amplitude discrimination thresholds in the ASD + ADHD group were higher than the typically developing control group ($t(401) = 2.97$, $p_{\text{Tukey}} = 0.017$; $d = 0.70$; $BF_{10} = 0.12.09$), while the ASD ($t(401) = 1.18$, $p_{\text{Tukey}} = 0.641$; $d = 0.34$; $BF_{10} = 0.39$) and ADHD ($t(401) = 1.08$, $p_{\text{Tukey}} = 0.701$; $d = 0.60$; $BF_{10} = 0.26$) groups did not. The ASD + ADHD group were also less accurate than controls ($t(358) = 2.65$, $p_{\text{Tukey}} = 0.041$; $d = 0.70$; $BF_{10} = 0.15.69$). See Supplementary Fig. 4(b)

With dual-site adaptation, there was also a significant main effect of group on amplitude discrimination thresholds ($F(3 , 292) = 6.69$, $p < 0.001$; $\eta^2_p = 0.06$, $BF_{10} = 9275192.92$). While there was no main effect of group on median reaction times ($F(3, 295) = 0.98$, $p = 0.402$; $\eta^2_p = 0.01$, $BF_{10} = 1.92$) or the number of reversals ($F(3, 268) = 1.13$, $p = 0.337$; $\eta^2_p = 0.01$, $BF_{10} = .21$), there was a main effect for accuracy ($F(3, 295) = 5.88$, $p < 0.001$; $\eta^2_p = 0.06$, $BF_{10} = 385978.44$). Similar to amplitude discrimination without adaptation and single-site adaptation, the posthoc comparisons found that individual's in the ASD + ADHD ($t(292) = 4.41$, $p_{\text{Tukey}} <$

0.001; $d = 0.70$; $BF_{10} = 0.644.83$) group had significantly higher amplitude discrimination thresholds with dual-site adaptation than the typically developing group, while those in the ASD ($t(292) = 1.00$, $p_{Tukey} = 0.748$; $d = 0.34$; $BF_{10} = 0.38$) and ADHD ($t(292) = 2.12$, $p_{Tukey} = 0.150$; $d = 0.60$; $BF_{10} = 1.78$) groups did not. Children in the ASD + ADHD group ($t(295) = 4.13$, $p_{Tukey} < 0.001$; $d = 0.70$; $BF_{10} = 251.53$) were also less accurate than those in the typically developing control group, while the ASD ($t(295) = 1.22$, $p_{Tukey} = 0.61$; $d = 0.34$; $BF_{10} = 0.48$) and ADHD ($t(295) = 1.92$, $p_{Tukey} = 0.220$; $d = 0.60$; $BF_{10} = 1.12$) group were not. See Supplementary Fig. 4(c)

Adaptation. When comparing the change in amplitude discrimination thresholds from the amplitude discrimination protocol to the amplitude discrimination with single-site adaptation and amplitude discrimination with dual-site adaptation protocols, there was a main effect of the single-site stimulus ($\chi2$ (1) = 41.32, $p < 0.001$), with participants showing higher (15.81 ± 2.41 μm) discrimination thresholds in the amplitude discrimination with single-site adaptation compared to the no adaptation ($t(408.82) = 6.58$, $p < 0.001$). There was no significant main effect of the dual-site adaptation stimulus ($\chi2$ (1) = 1.39, $p < .239$). Indeed, the increase in discrimination thresholds was only 2.31 ± 1.95 μm in the dual-site adaptation protocol compared to the no adaptation protocol (~85% less of an effect than the single-site adapting stimulus). There were no protocol by group interaction effects for either single ($\chi2$ (3) = 0.57, $p = 0.903$) or dual-site adaptation ($\chi2$ (3) = 0.57, $p = 0.903$) conditions, suggesting the change in thresholds (i.e., degree of adaptation) in both protocols was comparable between the groups. See Supplementary Fig. 4(d) and (e).

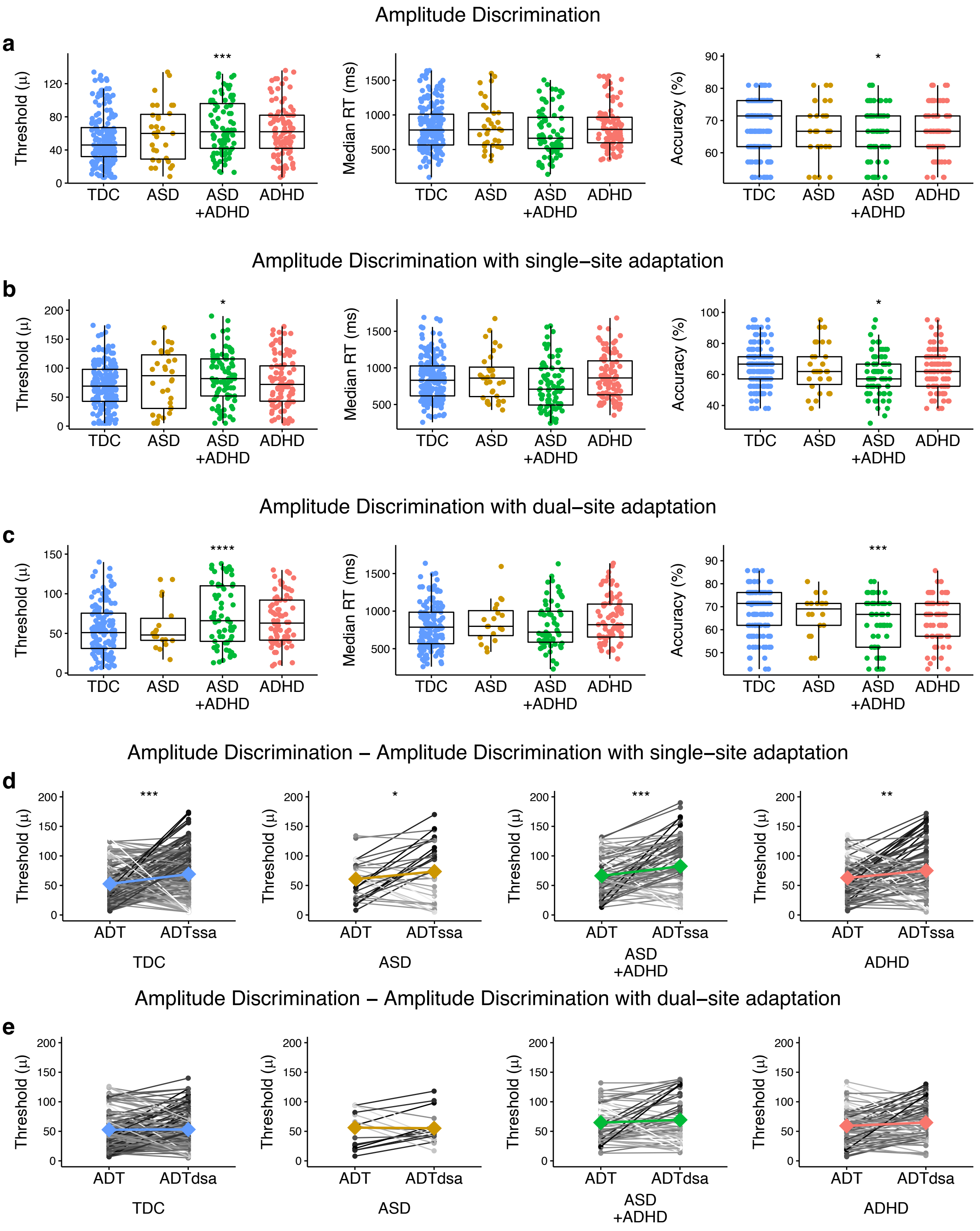

Supplementary Fig. 5. (**a**) Individual data points with overlaid boxplots visualizing the group comparisons on key performance outcomes on the amplitude discrimination (**b**) amplitude discrimination with single-site adaptation

protocols (**c**) and amplitude discrimination with dual-site adaptation protocols. (**d**) Individual data points and connecting slopes for discrimination thresholds on the amplitude discrimination with single-site adaptation and (**e**) amplitude discrimination with dual-site adaptation protocols for controls, the ASD, ASD + ADHD and ADHD groups. It is clear from the first plot of row that amplitude discrimination thresholds were higher for those with ASD and ASD + ADHD compared to controls and ADHD. There is evidence for an effect of protocol on amplitude discrimination thresholds, such that they became higher with single and dual-site adaptation. It is clear that the effect of the single-site adaptation stimulus has a more consistent impact on discrimination thresholds than the dual-site adaptation stimulus. There is not however, much evidence for group differences when comparing the impact of either the inclusion of the single-site or dual-site adaptation. Note that all statistical analyses presented in the main text of the manuscript for the plots presented in this figure are age corrected. Values in the plots presented within this figure are not corrected for age due to the loss of interpretability of units of measurement that occurs when presenting residuals rather than raw values. The precise number of participants in each group and further descriptive statistics can be found in Supplementary Methods - Supplementary Table 1. TDC = typically developing controls, ASD = autism spectrum disorders, ADHD = attention-deficit hyperactivity disorder, RT = reaction times, ADT = amplitude discrimination threshold; ADTssa = amplitude discrimination threshold with single-site adaptation; ADTdsa = amplitude discrimination threshold with dual-site adaptation.

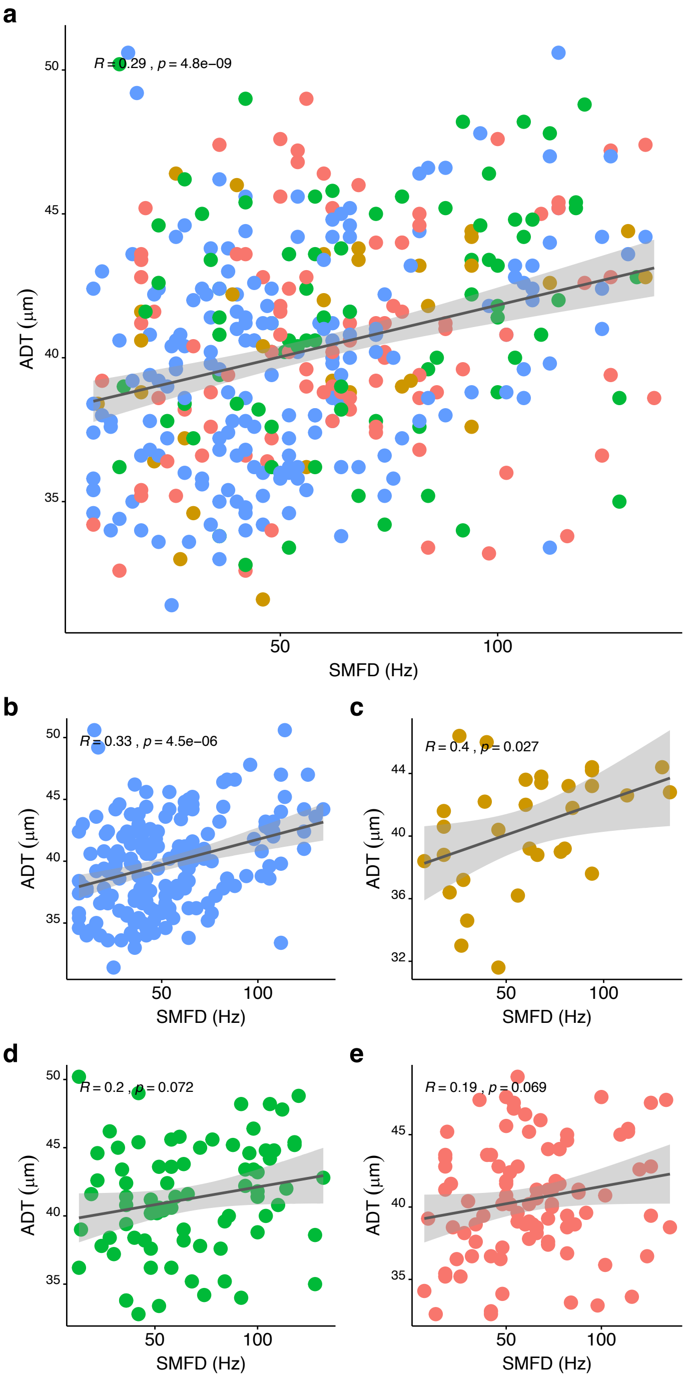

Supplementary Fig. 6. Correlations between amplitude discrimination and Simultaneous Frequency Discrimination thresholds. Plots are shown at the total sample level (**b**) and in just controls (**b**), ASD, (**c**) ASD + ADHD and (**d**) ADHD. While some correlations are not significant, this would likely be due to power, since the correlation at the total sample level is significant, with an effect that is comparable to those seen at the independent

groups level. TDC = typically developing controls, ASD = autism spectrum disorders, ADHD = attention-deficit hyperactivity disorder, ADT = amplitude discrimination threshold ; SMFD = simultaneous frequency discrimination

Temporal order judgement with carrier and stimulus-driven synchronization. Finally, we tested for an additional adaptation process which can be referred to as 'stimulus-driven synchronization[2]. In an early investigation, Tommerdahl and colleagues[2] found that having a constant low amplitude 'carrier' stimulus being concurrently delivered while participant performed temporal order judgement of sequentially delivered tactile stimuli would degrade a participant ability to judge the order in which those stimuli were received. This was suggested to reflect synchronization of the cortical ensembles that were otherwise required for tactile perception of each stimulation site independently. Subsequent work found that those with ASD were not affected by a low amplitude carrier stimulus, suggesting potential underconnectivity between local cortical sites in those with ASD[2]. Here, we also for stimulus-driven synchronization (i.e., connectivity between cortical sites) in children with ASD, ASD + ADHD, ADHD and controls using a modified version of our Temporal Order Judgement protocol, referred to as the Temporal Order Judgement protocol with carrier. The temporal order judgement with carrier protocol (See Supplementary Fig. 1(f)) was identical to the temporal order judgement protocol, except here we introduced a concurrent carrier stimulus (delivered for the duration of each trial.

With the inclusion of constant low amplitude carrier stimulus, there was a non-significant trend towards a main effect of group on temporal order judgement thresholds ($F(3, 259) = 1.89$, $p = 0.132$; $\eta^2_p = 0.02$, $BF_{10} = 2546.31$) and accuracy ($F(3, 247) = 1.87$, $p = 0.135$; $\eta^2_p = 0.02$, $BF_{10} = 2731.47$). There were no main effects of group on median reaction times ($F(3, 262) = 1.42$, $p = 0.238$; $\eta^2_p = 0.02$, $BF_{10} = 1.19$) or the number of reversals ($F(3, 279) = 1.43$, $p =$

0.232; $\eta^2_p = 0.02$, $BF_{10} = .03$). Like performance on the temporal order judgement protocol, individuals in the ASD + ADHD group showed a non-significant trend ($t(259) = 0.212$, $p_{Tukey} = 0.150$; $d = 0.70$; $BF_{10} = 1.29$) towards higher temporal order judgement thresholds than the typically developing control group when presented with a carrier stimulus. Conversely, there clearly a lack of a clear significant or meaningful difference between temporal order judgement thresholds between the ASD ($t(259) = -.53$, $p_{Tukey} = 0.950$; $d = 0.34$; $BF_{10} = 0.30$) and ADHD groups ($t(260) = 0.96$, $p_{Tukey} = 0.772$; $d = 0.59$, $BF_{10} = 0.26$). See Supplementary Fig. 6(b)

Stimulus-driven synchronization. When comparing temporal order judgment thresholds between protocols with and without the carrier stimulus, there was a significant main effect of condition on order judgement thresholds ($\chi 2$ (1) = 76.12, $p < 0.001$), with order judgement thresholds being 32.37 ± 3.44 ms higher when the stimuli were delivered with a carrier, this finding suggests the inclusion of the carrier stimulus had indeed induced synchronization between the cortical sites being stimulated. Interestingly, there was no condition by group interaction effect ($\chi 2$ (3) = 1.12, $p < .771$), suggesting that while the delivery of a low amplitude carrier stimulus did indeed degrade order judgement performance, presumably due to synchronization of cortical ensembles, its effect on performance is comparable between groups, suggesting comparable local connectivity in atypical neurodevelopment to typically developing controls. See Supplementary Fig. 6(c)

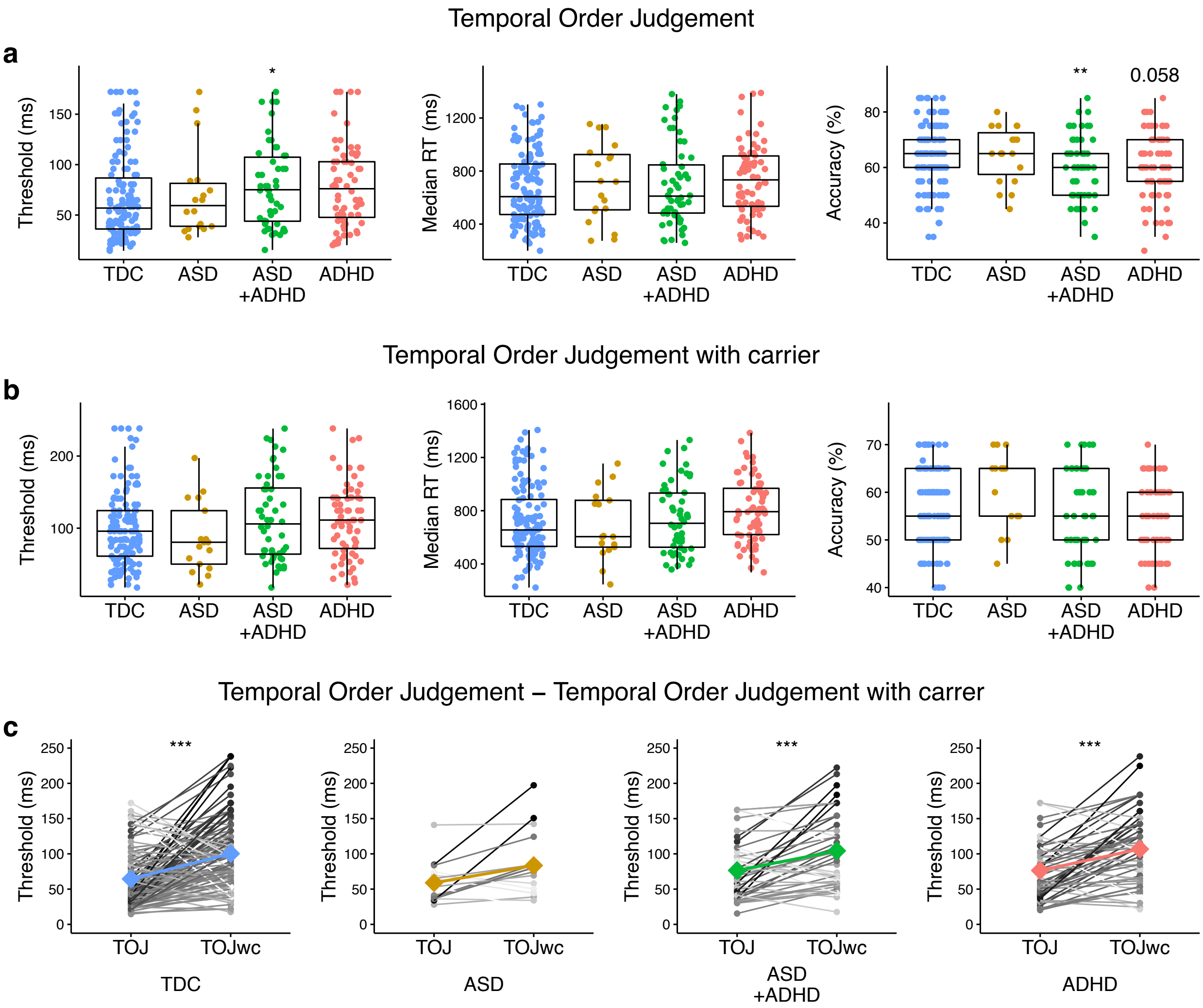

Supplementary Fig. 6. Individual data points with overlaid boxplots visualizing the group comparisons on key performance outcomes on the Temporal Order Judgement, including **(a)** Temporal Order Judgement Threshold, **(b)** Reaction Time and **(c)** Accuracy. While it would appear that only the ASD + ADHD and ADHD groups had elevated Temporal Order Judgement Thresholds compared to the typically developing control group, after controlling for age, only the ASD + ADHD group had higher Temporal Order Judgement Thresholds compared to controls. However, when looking at accuracy, the ASD + ADHD and ADHD group showed significant and a non-significant trend towards being less accurate than the typically developing control group (respectively). Note that all statistical analyses presented in the main text of the manuscript for the plots presented in this figure are age corrected. Values in the plots presented within this figure are not corrected for age due to the loss of interpretability of units of measurement that occurs when presenting residual rather than raw values. TDC = typically developing

controls, ASD = autism spectrum disorders, ADHD = attention-deficit hyperactivity disorder, TOJ = temporal order judgement, TOJwc = temporal order judgement with carrier

The following figures show the results of the correlation analyses that were included in the main body of the manuscript, as well as those that were not. The latter are the correlations between sequential frequency discrimination thresholds, amplitude discrimination with single- and dual-site adaptation thresholds and temporal order judgement with carrier thresholds to the subscales of the Sensory Processing Measure and Sensory Experience Questionnaire (see Supplementary Fig. (7)), ASD-specific symptomology (see Supplementary Fig. (8)) and ADHD-specific symptomology (see Supplementary Fig. (9)).

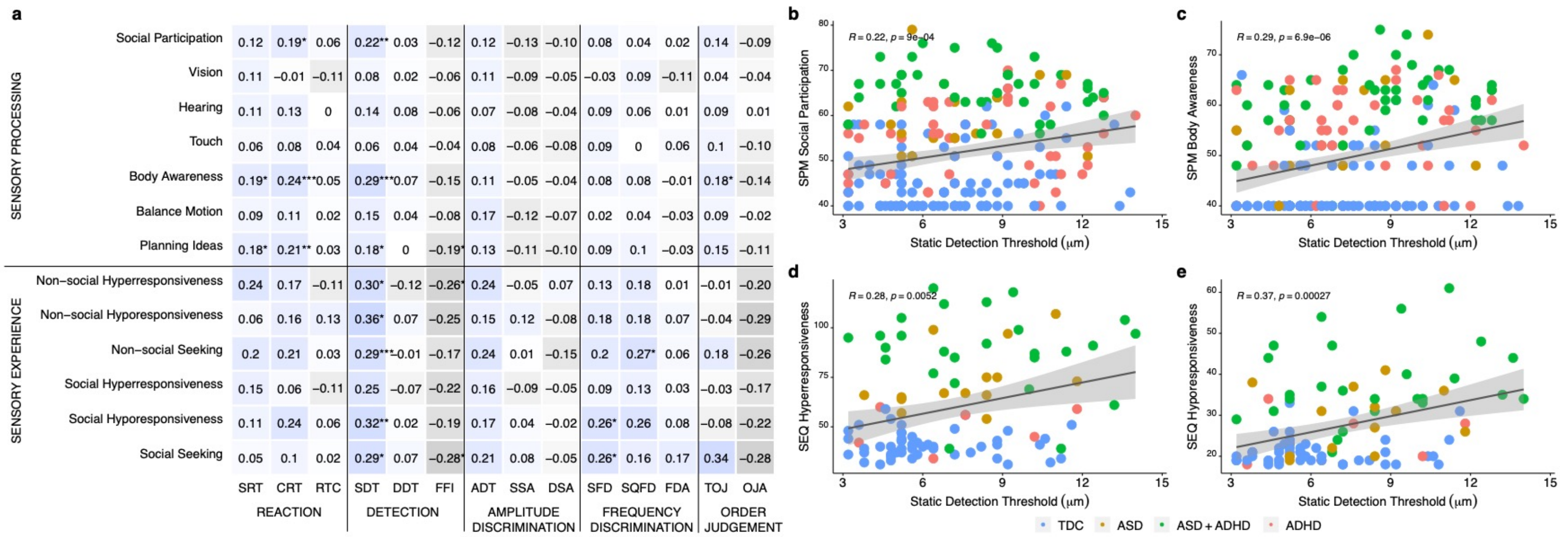

Supplementary Fig. 7. (a). Heatmap correlations between subscales of the Sensory Processing Measure and Sensory Experience Questionnaire (y-axis) and relevant tactile sensitivity metrics across each subdomain (x-axis). The '*' are used to denote statistical significance after correcting for multiple correlations using Bonferroni's correction (* = $p < .05$, ** = $p < .01$, *** = $p < .001$). 't' was used to denote a non-significant trend (i.e., $p < .10$). Due to the large number of correlations being conducted, Bonferroni corrections were applied to each of the correlation analyses conducted by multiplying the resulting *p*-value of each correlation between a given tactile sensitivity metric by the number of subscale items within each questionnaire. For example, for all of the correlations conducted between the Sensory Processing Measure and Static Detection Thresholds, the resulting p-value was multiplied by 7 (i.e., number of subscale items within the Sensory Processing Measure). It is clear that the tactile sensitivity metric that was most often associated with the subscales items of the Sensory Processing Measure and Sensory Experience Questionnaire questionnaires was Static Detection Thresholds. On the right we highlight the correlations between Static Detection Thresholds and (**b**)**.** social participation, (**c**)**.** body awareness, (**d**)**.** hyper-responsiveness and (**e**)**.** hypo-responsiveness. Note that the scatterplots presented in **d.** and **e.** are using combined scores of social and non-social hyper-responsiveness and hypo-responsiveness (which are otherwise presented

individually on the heatmap in **a.**). Note that the p-values presented within each scatterplot are the unadjusted p-values. TDC = typically developing controls, ASD = autism spectrum disorders, ADHD = attention-deficit hyperactivity disorder, SPM = sensory processing measure, SEQ = sensory experience questionnaire, SRT = simple reaction time, CRT = choice reaction time, RTC = reaction time change (between simple and choice), SDT = static detection threshold, DDT = Dynamic Detection Threshold, FFI = feedforward inhibition (difference in detection thresholds between dynamic and static), ADT = amplitude discrimination threshold, SSA = ADT with single-site adaptation, DSA = ADT with dual-site adaptation, SFD = simultaneous frequency discrimination, SQFD = sequential frequency discrimination, FDA = frequency discrimination adaptation (difference in frequency discrimination thresholds between sequential and simultaneous frequency discrimination), TOJ = temporal order judgement, OJA = order judgement adaptation (difference in order judgement thresholds with and without carrier stimulus).

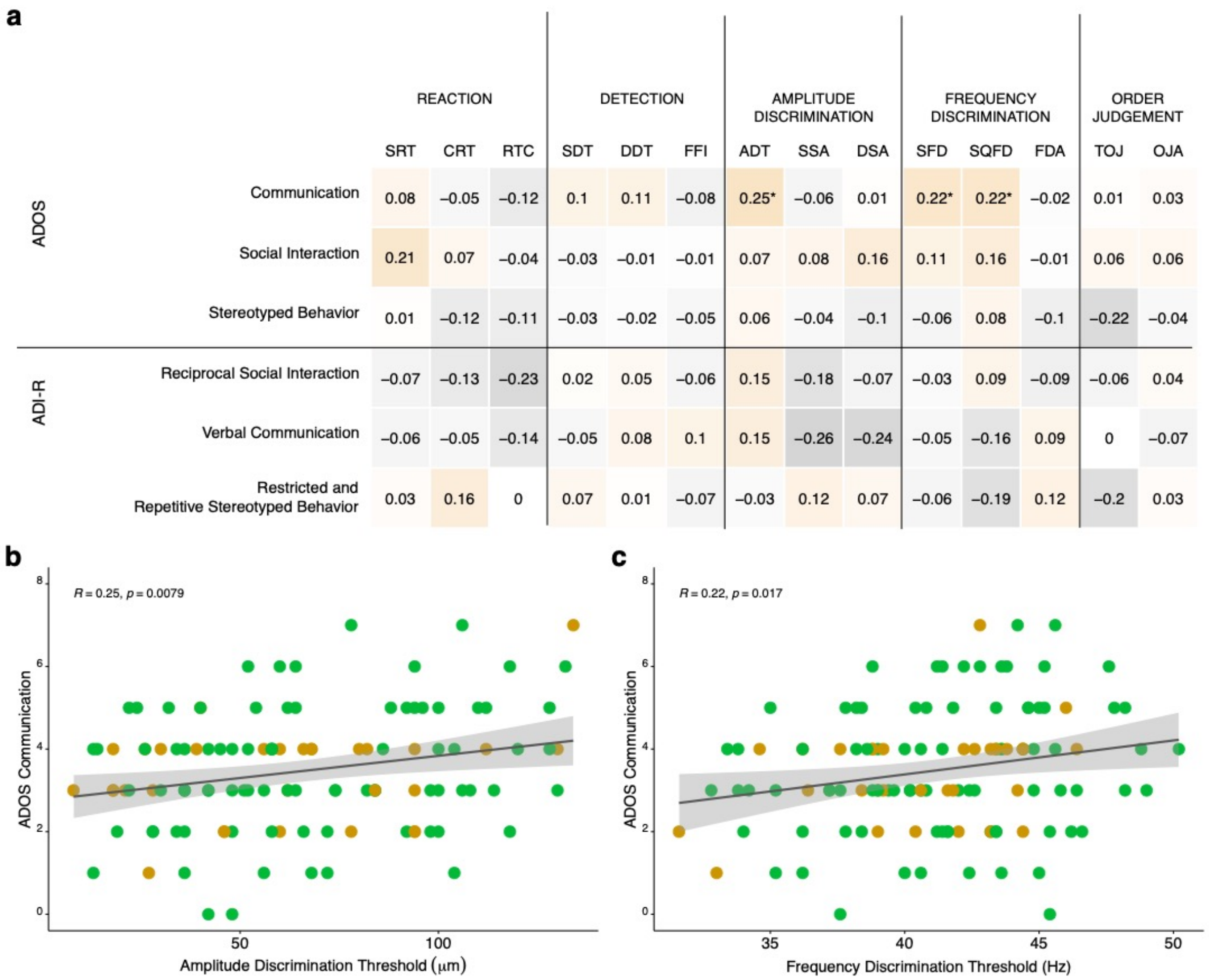

Supplementary Fig. 8. (a). Heatmap correlations between subscales of the Autism Diagnostic Observation Scale and Autism Diagnostic Interview-Revised (y-axis) and relevant tactile sensitivity metrics (x-axis). The '*' are used to denote statistical significance after correcting for multiple correlations using Bonferroni's correction (* = $p < .05$, ** = $p < .01$, *** = $p < .001$). 't' was used to denote a non-significant trend (i.e., $p < .10$). Due to the large number of correlations being conducted, Bonferroni corrections were applied to each of the correlation analyses conducted by multiplying the resulting *p*-value of each correlation between a given tactile sensitivity metric by the number of subscale items within each questionnaire (see the figure caption of Fig. 7. for an example). Two associations survived Bonferroni corrections and are presented in the bottom row of the figure. There were positive correlations between problems with Communication on the Autism Diagnostic Observation Scale with (**b**). Amplitude Discrimination Thresholds and (**c**). Frequency Discrimination Thresholds. Note that the p-values presented within each scatterplot are the unadjusted p-values. ASD = autism spectrum disorders, ADHD = attention-deficit

hyperactivity disorder, ADOS = Autism Diagnostic Observation Scale, ADI-R = Autism Diagnostic Interview – Revised, SRT = simple reaction time, CRT = choice reaction time, RTC = reaction time change (between simple and choice), SDT = static detection threshold, DDT = Dynamic Detection Threshold, FFI = feedforward inhibition (difference in detection thresholds between dynamic and static), ADT = amplitude discrimination threshold, SSA = ADT with single-site adaptation, DSA = ADT with dual-site adaptation, SFD = simultaneous frequency discrimination, SQFD = sequential frequency discrimination, FDA = frequency discrimination adaptation (difference in frequency discrimination thresholds between sequential and simultaneous frequency discrimination), TOJ = temporal order judgement, OJA = order judgement adaptation (difference in order judgement thresholds with and without carrier stimulus).

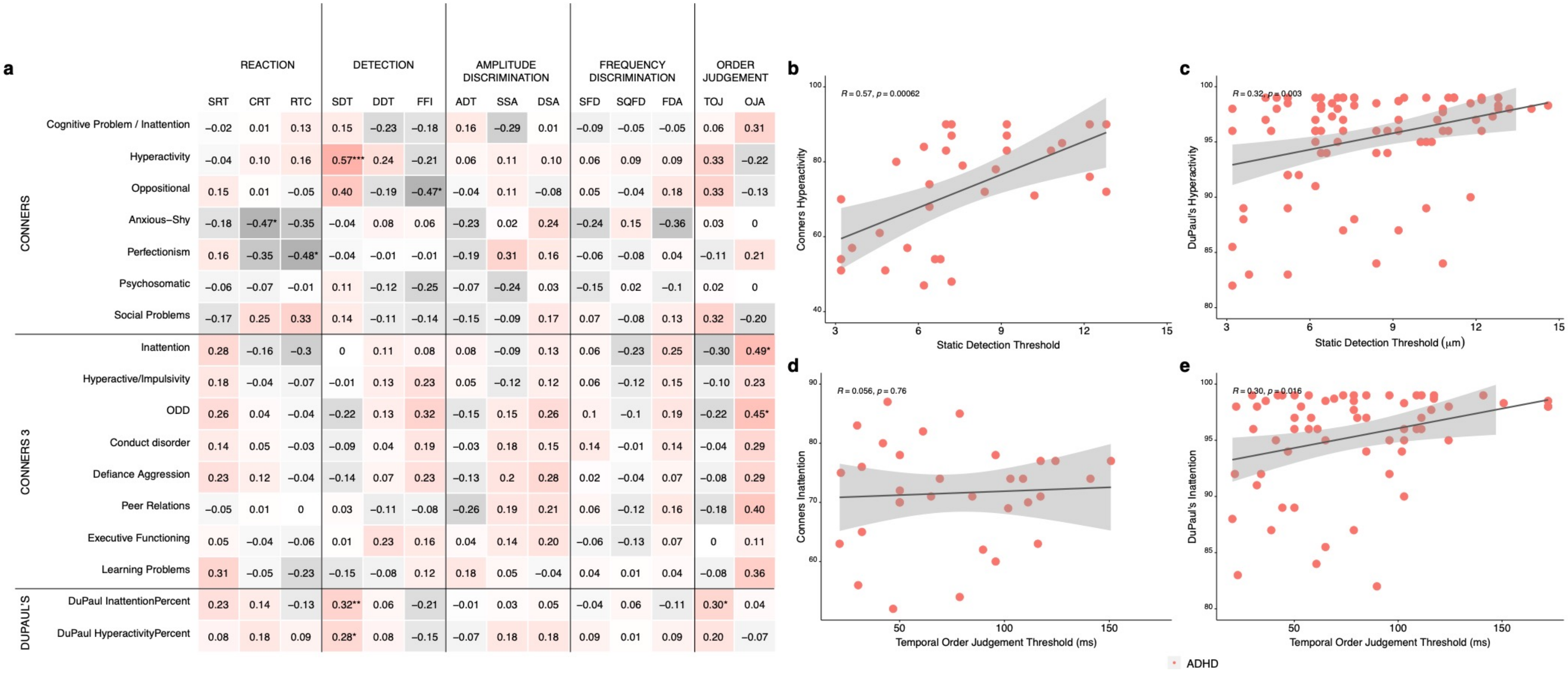

Supplementary Fig. 9 (a). Heatmap correlations between subscales of the Conners, Conners 3 and DuPaul's ADHD parent rating scales (y-axis) and relevant tactile sensitivity metrics (x-axis). The '*' are used to denote statistical significance after correcting for multiple correlations using Bonferroni's correction (* = $p < .05$, ** = $p < .01$, *** = $p < .001$). 't' was used to denote a non-significant trend (i.e., $p < .10$). Due to the large number of correlations being conducted, Bonferroni corrections were applied to each of the correlation analyses conducted by multiplying the resulting *p*-value of each correlation between a given tactile sensitivity metric by the number of subscale items within each. There were positive correlations between (**a**) Hyperactivity subscales on the Conners and (**c**) DuPaul's with Static Detection Thresholds. While there was no significant association between Inattention on the Conners subscale (**d**), there was a significant

positive correlation between DuPaul's inattention and Temporal Order Judgement thresholds (**e**). Note that the p-values presented within each scatterplot are the unadjusted p-values. ADHD = attention-deficit hyperactivity disorder, ODD = oppositional defiant disorder, SPM = sensory processing measure, SEQ = sensory experience questionnaire, SRT = simple reaction time, CRT = choice reaction time, RTC = reaction time change (between simple and choice), SDT = static detection threshold, DDT = Dynamic Detection Threshold, FFI = feedforward inhibition (difference in detection thresholds between dynamic and static), ADT = amplitude discrimination threshold, SSA = ADT with single-site adaptation, DSA = ADT with dual-site adaptation, SFD = simultaneous frequency discrimination, SQFD = sequential frequency discrimination, FDA = frequency discrimination adaptation (difference in frequency discrimination thresholds between sequential and simultaneous frequency discrimination), TOJ = temporal order judgement, OJA = order judgement adaptation (difference in order judgement thresholds with and without carrier stimulus).

When compared to typically developing controls, amplitude and frequency discrimination thresholds were found to be significantly higher for those in the ASD + ADHD group, while being comparable (to typically developing controls) in the ASD and ADHD groups. When collapsing across groups, discrimination threshold (and the relevant adaptation metrics: single-site adaptation and dual-site adaptation) were significantly predictive of ASD but not ADHD-related clinical symptoms. While the results from the group comparisons would suggest a potential additive or interactive effect of having comorbid ASD + ADHD (since those in the ASD and ADHD groups did not have significantly higher discrimination thresholds than those in the typically developing control group), the correlational analyses would suggest that difficulties with discrimination are more specifically related to symptoms or pathophysiology of ASD. We further investigated this idea by running group-level analyses (i.e., separate correlations for those in the ASD and ASD + ADHD groups) for the correlations between tactile discrimination thresholds and ASD-related symptoms that were found to be significant (e.g., amplitude discrimination, and sequential and simultaneous frequency discrimination). In support of our interpretation, the correlations between amplitude, simultaneous frequency and sequential frequency discrimination thresholds were all stronger than the correlations presented in Fig. 7 (b, c and d) of the main manuscript when the sample was constrained to only those in the ASD group (See Supplementary Fig. 9). The correlations when only including the ASD group were also stronger than the correlations when the sample when constrained to only those in the ASD + ADHD group.

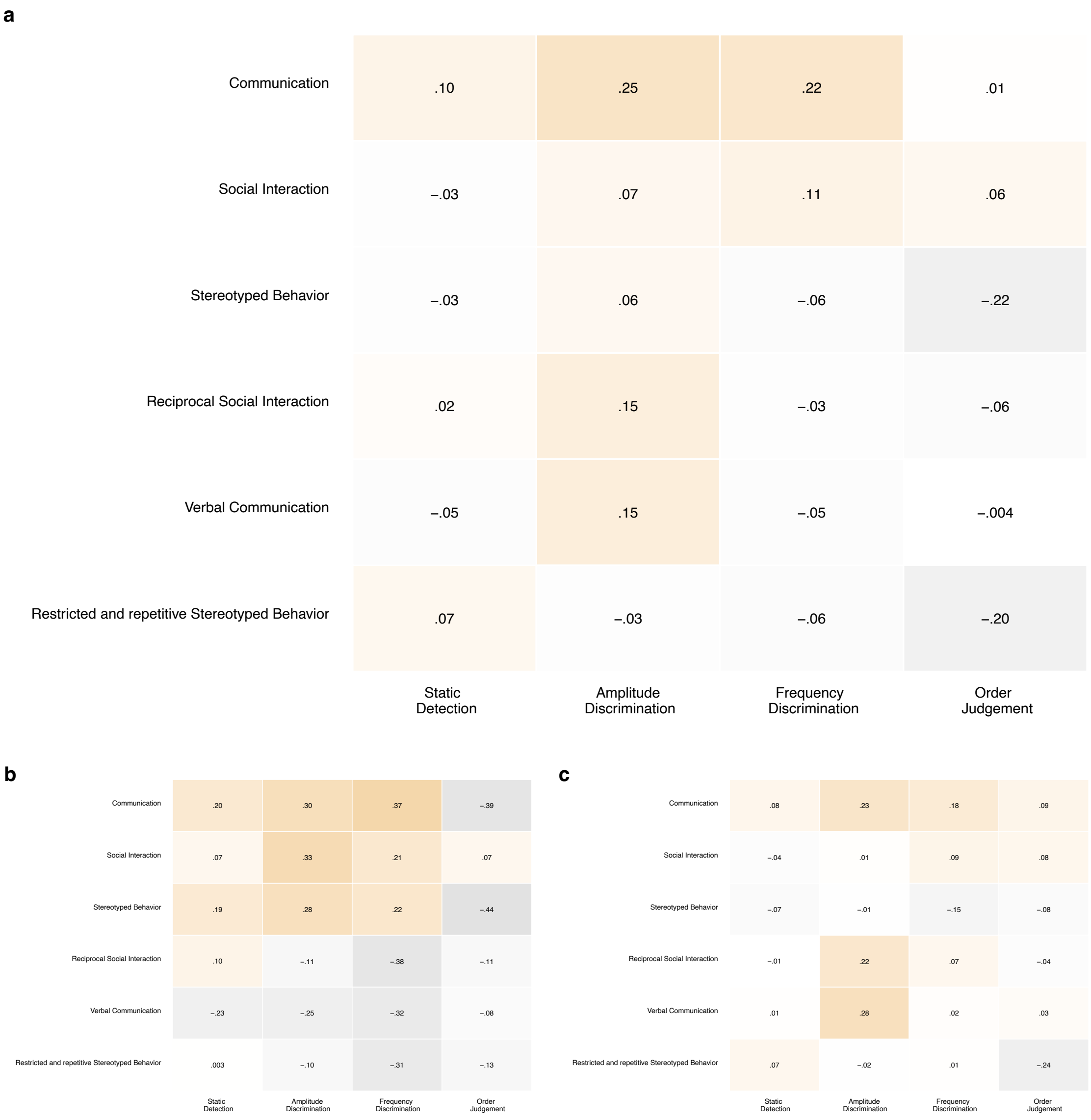

Supplementary Fig. 10. Heatmap correlations between subscales of the Sensory Processing Measure and Sensory Experience Questionnaire (y-axis) and relevant tactile sensitivity metrics across each subdomain (x-axis). (**a**) refers to the heatmap correlation plot presented in the main body of the manuscript, which contained both children who criteria for ASD, as well as those who met criteria for ASD + ADHD. (**b**) contains the same correlation as (a), however, (b) only includes children who had a primary diagnosis of ASD, who did not also have a diagnosis of ASD + ADHD. Finally, (**c**) contains the same correlations as (a) and (b), but only include those in the ASD + ADHD group. As is discernible, the correlations between the tactile sensitivity thresholds are *strongest* in (b).

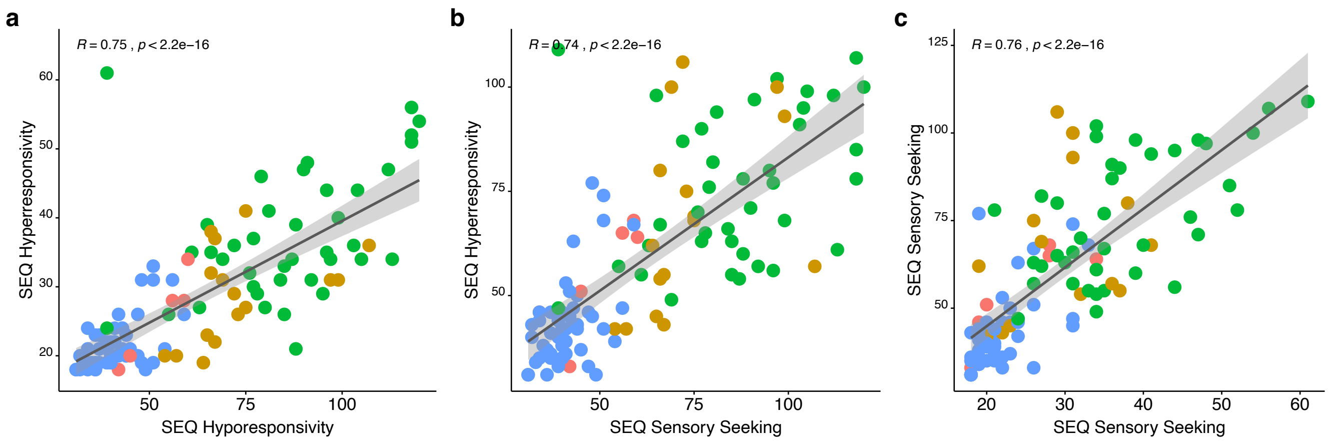

Supplementary Fig. 11. Plots depicting the linear associations between Sensory Hyper-responsivity, Sensory Hypo-responsivity and Sensory Seeking scores from the Sensory Experience Questionnaire. As can be discerned, all three outcome measures correlate strongly with each other, suggesting that those scoring high on one outcome also score high on the others. For example, a child that is hyper-responsive is also likely to be a child that is hypo-responsive. SPM = sensory processing measure, SEQ = sensory experience questionnaire.

Supplementary Table 1. Descriptive statistics for each vibrotactile task by group

	TDC			ASD			ASD + ADHD			ADHD		
**Reaction Time**	N	M	SD	N	M	SD	N	M	SD	N	M	SD
Simple												
Median RT (ms)	195	343.24	113.81	31	352.44	128.75	91	376.25	117.9	97	383.06	119.01
RT variability (ms)	173	101.66	51.87	24	105.96	43.4	74	108.9	56.18	70	138.94	68.16
Choice												
Median RT (ms)	189	702.13	188.22	32	732.33	232.62	85	763.66	217.18	98	791.02	183.56
RT variability (ms)	168	235.89	131.71	23	204.4	138.56	65	299.78	167.41	70	313.53	173.72
Accuracy (%)	185	94.38	5.32	31	93.55	6.35	78	92.5	6.53	85	93.71	6.42
**Detection**	N	M	SD	N	M	SD	N	M	SD	N	M	SD
Static												
Median RT (ms)	191	537.01	210.93	32	577.89	193.33	86	622.37	247.27	98	650.33	221.02
Accuracy (%)	185	90.32	5.74	32	89.71	5.6	75	87.61	6.39	86	87.45	6.81
Reversals (N)	195	3.23	2.07	33	3.42	1.95	95	4.75	2.77	103	4.29	2.28
Threshold (μ)	184	6.63	2.34	32	7.36	2.33	76	8.11	2.88	88	8.2	2.93
Dynamic												
Median RT (ms)	186	5152.63	1848.14	32	5376.38	1827.33	86	5398.57	2461	93	4824.63	1923.84
Accuracy (%)	192	86.83	17.17	32	89.28	14.96	87	82.76	18.88	93	81.57	18.31
Threshold (μ)	186	9.04	3.04	32	9.23	3.42	80	9.53	4.01	91	8.69	3.32
**Amplitude Discrimination**	N	M	SD	N	M	SD	N	M	SD	N	M	SD
Without adaptation												
Median RT (ms)	184	829.98	320.89	34	849.51	347.9	81	743.48	332.35	96	831.36	308.83
Accuracy (%)	173	68.43	7.58	29	67.32	8.51	74	66.15	8.02	87	67.49	7.09
Reversals (N)	169	6.49	1.71	33	7	1.56	78	6.9	1.56	91	6.81	1.65
Threshold (μ)	187	53.13	30.89	31	60.68	33.95	85	67.19	32.36	97	63.04	31.21
With single-site adaptation												
Median RT (ms)	184	857.59	304.43	32	882.41	322.22	81	765.92	329.06	93	877.78	303.65

Accuracy (%)	188	64.79	12.36	34	64.15	15.25	85	59.94	12.84	99	62.96	13.22
Reversals (N)	188	6.44	2.55	34	6.5	3.02	87	6.84	2.41	99	6.82	2.44
Threshold (μ)	188	69.58	38.6	34	78.41	49.94	85	84.86	43.85	99	75.88	43.67
With dual-site adaptation												
Median RT (ms)	144	810.65	297.22	19	860.58	275.56	61	819.16	312.79	76	892.23	310.5
Accuracy (%)	144	68.25	9.51	18	66.4	9.31	61	63.23	11.1	77	64.97	9.29
Reversals (N)	129	6.61	1.74	17	6.65	1.5	55	6.82	1.72	72	7.1	1.7
Threshold (μ)	142	54.63	31.45	18	58.67	30.45	61	72.34	38.2	76	66.63	30.9
**Frequency Discrimination**	N	M	SD	N	M	SD	N	M	SD	N	M	SD
Simultaneous												
Median RT (ms)	180	813.28	319.31	32	756.38	234.02	83	738.78	344.06	87	869.79	327.48
Accuracy (%)	187	55.72	10.41	33	54.83	9.53	86	51.72	11.22	98	54.08	11.16
Reversals (N)	174	7.82	1.63	30	8.07	1.41	81	7.98	1.56	92	7.99	1.59
Threshold (Hz)	187	39.86	3.74	33	40.61	3.66	86	41.47	4.07	98	40.71	3.96
Sequential												
Median RT (ms)	182	694.47	255.06	30	676.67	246.16	75	685.97	257	91	781.01	280.24
Accuracy (%)	182	62.12	11.13	31	62.42	12.57	84	58.57	10.69	97	59.59	12.18
Reversals (N)	165	7.87	1.72	29	7.69	1.63	76	7.88	1.8	90	8.03	1.58
Threshold (Hz)	182	38.71	3.72	31	39.05	4.16	83	39.79	3.52	95	39.19	3.77
**Order Judgement**	N	M	SD	N	M	SD	N	M	SD	N	M	SD
Without carrier												
Median RT (ms)	135	673.94	262.34	19	708.87	292.04	58	698.66	302.83	70	734.8	268.67
Accuracy (%)	140	64.69	11.2	19	64.74	9.93	59	59.49	11.01	75	60	11.65
Reversals (N)	120	8.5	1.74	18	8.78	1.35	56	8.89	1.45	63	9.03	1.76
Threshold (ms)	131	67.09	41.72	18	70.53	43.14	50	79.27	40.73	66	78.33	40.26
With carrier												
Median RT (ms)	128	720.78	266.93	17	690.18	257.96	54	742.62	258.48	68	801.19	239.77
Accuracy (%)	120	56.68	7.86	15	60.33	8.12	53	55.66	8.94	64	54.53	7.39
Reversals (N)	134	9.49	2.15	19	8.32	2	58	9.41	2.49	73	9.42	2.27

Threshold (ms)	128	100.19	51.47	17	86.96	48.56	52	113.05	58.46	67	111.58	51.14

Note: The values presented in this table are those estimated after the removal of outliers using the median absolute deviation method described in the Methods. The sample sizes here are consistent to the data presented in Fig. 2 to 5. TDC = typically developing controls, ASD = autism spectrum disorders, ADHD = attention-deficit hyperactivity disorder. RT = reaction times. N = number of participants, M = mean, SD = standard deviation.

Supplementary Discussion

As is always the case for group comparisons on behavioral tasks, the identification of group differences does not necessarily suggest alterations or deficit that are homogeneous across the group (e.g., not all children in the ASD group had elevated discrimination thresholds relative to those in the typically developing control group). Therefore, we have presented individual data points throughout our figures. For instance, while the group differences in static detection thresholds in Fig. 2a are discernible when viewing the boxplots, it is also apparent that there is substantial overlap of individual data points between groups. This finding is not just constrained to the present study but is widely discussed in psychology and the cognitive neurosciences[3]. We highlight this point as a limitation in the current study since despite suggesting disorder-shared (static detection thresholds in ASD, ADHD and ASD + ADHD) and disorder-specific (amplitude discrimination in ASD and temporal order judgement in ADHD) alterations, it is clear that not all individuals with ASD have difficulties with amplitude discrimination and not all those with ADHD have difficulties with temporal order judgement. What these results do suggest is that individuals with ASD, ADHD and ASD + ADHD are more likely than typically developing controls to have difficulties with either static detection, amplitude discrimination or order judgement.

Possible explanations for the degree of overlap across these tactile sensitivity metrics is that despite the consistent finding of group differences for the same or similar tactile sensitivity metrics in other studies, there is inherent and perhaps unavoidable measurement error when assessing sensory processes. Another possible and non-mutually exclusive explanation is that there really are just a large number of individuals who meet diagnosis for a given neurodevelopmental disorder who do not also have the sensory alterations that these group

averages would suggest. Future efforts to either reduce measurement error or to increase the sensitivity of sensory and behavioral measures will help to overcome some of the issues that we are currently faced with in individual differences research. Recent work[4] has suggested that elevated thresholds (measured using Von Frey hairs) in ASD are due to altered perceptual decision-making rather than actual elevated thresholds. Our results do not show elevated thresholds in our ASD-only cohorts, nor does this explain elevations in discrimination threshold. It could be, however, that increased trial-to-trial noise affects threshold measures. Additional higher-order cognitive function, or the examination of response criterions, trial-by-trial variability and dynamic range, should be taken into account when examining perception in ASD.

Finally, as is common in psychology and cognitive neuroscience, the group differences in tactile sensitivity we identified in the neurodevelopmental disorders are described as being an abnormality or deficit. While it is true that these differences are related to some of the behavioral and social problems experienced by those diagnosed with ASD, ADHD or ASD + ADHD (as our results would suggest), it is also worth considering these differences from the perspective of contextual strengths and weaknesses. Indeed, there have been many studies in which altered sensory sensitivity has been linked to better task performance in those with ASD[5–7]. Similarly, in ADHD, having higher temporal order judgement thresholds (or a wider temporal binding window) could potentially be more adaptive in certain contexts.

Supplementary Table 2: Group Comparisons on SPM and SEQ scale items

Measures	Groups		
	ADHD	ASD	ASD + ADHD
**SPM Total Social Participation Score**			
ASD	$p = 0.015$	-	-
ASD + ADHD	$p < 0.0001$	$p < 0.0001$	-
Control	$p < 0.0001$	$p < 0.0001$	$p < 0.0001$
**SPM Total Vision Score**			
ASD	$p < 0.001$	-	-
ASD + ADHD	$p < 0.0001$	$p < 0.0001$	-
Control	$p < 0.0001$	$p < 0.0001$	$p < 0.0001$
**SPM Total Hearing Score**			
ASD	$p < 0.01$	-	-
ASD + ADHD	$p < 0.0001$	$p < 0.001$	-
Control	$p < 0.0001$	$p < 0.0001$	$p < 0.0001$
**SPM Total Touch Score**			
ASD	$p < 0.001$	-	-
ASD + ADHD	$p < 0.0001$	$p = 0.024$	-
Control	$p < 0.0001$	$p < 0.0001$	$p < 0.0001$
**SPM Total Sensory Systems Score**			
ASD	$p < 0.01$	-	-
ASD + ADHD	$p < 0.0001$	$p < 0.01$	-
Control	$p < 0.0001$	$p < 0.0001$	$p < 0.0001$
**SPM Total Body Awareness Score**			
ASD	$p < 0.0001$	-	-
ASD + ADHD	$p < 0.0001$	$p < 0.01$	-
Control	$p < 0.0001$	$p < 0.0001$	$p < 0.0001$
**SPM Total Balance Score**			
ASD	$p < 0.0001$	-	-
ASD + ADHD	$p < 0.0001$	$p < 0.001$	-
Control	$p < 0.0001$	$p < 0.0001$	$p < 0.0001$
**SPM Total Planning Ideas Score**			
ASD	$p = .350$	-	-
ASD + ADHD	$p < 0.0001$	$p < 0.001$	-
Control	$p < 0.0001$	$p < 0.0001$	$p < 0.0001$
**SEQ Raw Hyper-responsiveness Score**			
ASD	$p < 0.01$	-	-
ASD + ADHD	$p < 0.0001$	$p = 0.017$	-
Control	$p < 0.01$	$p < 0.0001$	$p < 0.0001$
**SEQ Raw Hypo-responsiveness Score**			
ASD	$p < 0.01$	-	-
ASD + ADHD	$p < 0.001$	$p < 0.0001$	-
Control	$p < 0.01$	$p < 0.01$	$p < 0.0001$

**SEQ Raw Sensory Seeking Score**			
ASD	p = .415	-	-
ASD + ADHD	p = 0.012	p = .533	-
Control	p = .532	p < 0.0001	p < 0.0001
**SEQ Raw Hyper-responsiveness Score (Social)**			
ASD	p = 0.0160	-	-
ASD + ADHD	p < 0.0001	p = 0.132	-
Control	p < 0.0001	p < 0.0001	p < 0.0001
**SEQ Raw Hypo-responsiveness Score (Social)**			
ASD	p < 0.0001	-	-
ASD + ADHD	p < 0.001	p < 0.001	-
Control	p < 0.0001	p < 0.01	p < 0.0001
**SEQ Raw Sensory Seeking Score (Social)**			
ASD	p < 0.0001	-	-
ASD + ADHD	p < 0.0001	p < 0.0001	-
Control	p = 0.143	p < 0.01	p < 0.0001
**SEQ Raw Hyper-responsiveness Score (Non-social)**			
ASD	p = 0.027	-	-
ASD + ADHD	p < 0.0001	p = .185	-
Control	p < 0.0001	p < 0.0001	p < 0.0001
**SEQ Raw Hypo-responsiveness Score (Non-social)**			
ASD	p < 0.0001	-	-
ASD + ADHD	p < 0.01	p < 0.01	-
Control	p < 0.0001	p < 0.01	p < 0.0001
**SEQ Raw Sensory Seeking Score (Non-social)**			
ASD	p = .269	-	-
ASD + ADHD	p < 0.01	p = .636	-
Control	p = 0.902	p < 0.0001	p < 0.0001

TDC = typically developing controls, ASD = autism spectrum disorders, ADHD = attention-deficit hyperactivity disorder. RT = reaction times, SPM = sensory processing measure, SEQ = sensory experience questionnaire

Supplementary References

1. Tannan, V., Simons, S., Dennis, R. G. & Tommerdahl, M. Effects of adaptation on the capacity to differentiate simultaneously delivered dual-site vibrotactile stimuli. *Brain Res.* (2007). doi:10.1016/j.brainres.2007.10.024
2. Tommerdahl, M., Tannan, V., Holden, J. K. & Baranek, G. T. Absence of stimulus-driven synchronization effects on sensory perception in autism: Evidence for local underconnectivity? *Behav. Brain Funct.* (2008). doi:10.1186/1744-9081-4-19
3. Szucs, D. & Ioannidis, J. P. A. Empirical assessment of published effect sizes and power in the recent cognitive neuroscience and psychology literature. *PLoS Biol.* (2017). doi:10.1371/journal.pbio.2000797
4. Quinde-Zlibut, J. M. *et al.* Elevated Thresholds for Light Touch in Children With Autism Reflect More Conservative Perceptual Decision-Making Rather Than a Sensory Deficit. *Front. Hum. Neurosci.* (2020). doi:10.3389/fnhum.2020.00122
5. Jones, E. J. H., Dawson, G. & Webb, S. J. Sensory hypersensitivity predicts enhanced attention capture by faces in the early development of ASD. *Dev. Cogn. Neurosci.* (2018). doi:10.1016/j.dcn.2017.04.001
6. Falter, C. M., Braeutigam, S., Nathan, R., Carrington, S. & Bailey, A. J. Enhanced access to early visual processing of perceptual simultaneity in autism spectrum disorders. *J. Autism Dev. Disord.* (2013). doi:10.1007/s10803-012-1735-1
7. Jones, C. R. G. *et al.* Auditory discrimination and auditory sensory behaviours in autism spectrum disorders. *Neuropsychologia* (2009). doi:10.1016/j.neuropsychologia.2009.06.015
