# Supplementary material for: Disorder-specific alterations of tactile sensitivity in neurodevelopmental disorders
Source: Commun Biol. 2021 Jan 22;4:97. doi: 10.1038/s42003-020-01592-y (PMC7822903; doi:10.1038/s42003-020-01592-y)
Supplement: Supplementary file 3 — Reporting Summary [file 42003_2020_1592_MOESM3_ESM.pdf]

nature research

Corresponding author(s): COMMSBIO-20-1858-T

Last updated by author(s): 18/11/20

Reporting Summary

Nature Research wishes to improve the reproducibility of the work that we publish. This form provides structure for consistency and transparency in reporting. For further information on Nature Research policies, see our Editorial Policies and the Editorial Policy Checklist.

Statistics

For all statistical analyses, confirm that the following items are present in the figure legend, table legend, main text, or Methods section.

n/a	Confirmed	
☐	☒	The exact sample size (*n*) for each experimental group/condition, given as a discrete number and unit of measurement
☐	☒	A statement on whether measurements were taken from distinct samples or whether the same sample was measured repeatedly
☐	☒	The statistical test(s) used AND whether they are one- or two-sided<br>*Only common tests should be described solely by name; describe more complex techniques in the Methods section.*
☐	☒	A description of all covariates tested
☐	☒	A description of any assumptions or corrections, such as tests of normality and adjustment for multiple comparisons
☐	☒	A full description of the statistical parameters including central tendency (e.g. means) or other basic estimates (e.g. regression coefficient) AND variation (e.g. standard deviation) or associated estimates of uncertainty (e.g. confidence intervals)
☐	☒	For null hypothesis testing, the test statistic (e.g. *F*, *t*, *r*) with confidence intervals, effect sizes, degrees of freedom and *P* value noted<br>*Give P values as exact values whenever suitable.*
☒	☐	For Bayesian analysis, information on the choice of priors and Markov chain Monte Carlo settings
☒	☐	For hierarchical and complex designs, identification of the appropriate level for tests and full reporting of outcomes
☒	☐	Estimates of effect sizes (e.g. Cohen's *d*, Pearson's *r*), indicating how they were calculated

Our web collection on statistics for biologists contains articles on many of the points above.

Software and code

Policy information about availability of computer code

Data collection	The code used to collect the tactile data was provided by Cortical Metrics (for use with the Cortical Metrics Stimulator)
Data analysis	The code used to process the vibrotactile data collected from the Cortical Metrics device are available from https://github.com/HeJasonL/BATD. The code used to analyze the data and generate the figures in this study are also available as an RMarkdown file on the Open Science Framework: https://osf.io/9mdc6/

For manuscripts utilizing custom algorithms or software that are central to the research but not yet described in published literature, software must be made available to editors and reviewers. We strongly encourage code deposition in a community repository (e.g. GitHub). See the Nature Research guidelines for submitting code & software for further information.

Data

Policy information about availability of data

All manuscripts must include a data availability statement. This statement should provide the following information, where applicable:

- Accession codes, unique identifiers, or web links for publicly available datasets
- A list of figures that have associated raw data
- A description of any restrictions on data availability

As provided in the data availability statement of the submitted manuscript, the raw data used in this study, which can be analyzed by the code provided in the links above is available upon request and will become available on the Open Science Framework upon acceptance of the manuscript.

Field-specific reporting

Please select the one below that is the best fit for your research. If you are not sure, read the appropriate sections before making your selection.

☐ Life sciences ☒ Behavioural & social sciences ☐ Ecological, evolutionary & environmental sciences

For a reference copy of the document with all sections, see nature.com/documents/nr-reporting-summary-flat.pdf

Behavioural & social sciences study design

All studies must disclose on these points even when the disclosure is negative.

Study description	The study is a quantitative experimental study investigating differences in tactile processing in typically developing children, children with Autism Spectrum Disorders (ASD), Attention-deficit Hyperactivity Disorders (ADHD) and ASD + ADHD combined.
Research sample	The research sample consisted of children that were between 8 and 12 years of age, with ASD with no co-diagnosis of ADHD (n = 45), ADHD with no co-diagnosis of ASD (n = 102), combined ASD + ADHD (n = 81) and typically developing controls (n = 191). The study sample was chosen due to tactile processing being commonly reported in children with these neurodevelopmental conditions.
Sampling strategy	Sampling was stratified and based on prior diagnosis. No specific sample sizes were chosen except for ~100 per group to be able to investigate diagnosis and sub-diagnosis, and are sufficient based on initial pilot studies. Sample sizes are large for studies of these kind. No criteria were used to terminate sampling and data were acquired as part of ongoing grants.
Data collection	Children were present for visits at multiple points (1 or 2 full day visits) of which qualitative testing (questionnaires) were performed with the parent as children performed cognitive testing. Cognitive testing was always accompanied by a research assistant. Research assistants were not blind to diagnosis as this is near impossible. However, researcher assistants were blind to diagnosis.
Timing	Data were collected between January 2012 and July 2019.
Data exclusions	Data include all participants who completed tactile testing
Non-participation	The data include all participants who completed tactile testing.
Randomization	Participants were allocated to groups based on diagnosis, and therefore grouping could not be randomized.

Reporting for specific materials, systems and methods

We require information from authors about some types of materials, experimental systems and methods used in many studies. Here, indicate whether each material, system or method listed is relevant to your study. If you are not sure if a list item applies to your research, read the appropriate section before selecting a response.

Materials & experimental systems

n/a	Involved in the study	
☒	☐	Antibodies
☒	☐	Eukaryotic cell lines
☒	☐	Palaeontology and archaeology
☒	☐	Animals and other organisms
☐	☒	Human research participants
☒	☐	Clinical data
☒	☐	Dual use research of concern

Methods

n/a	Involved in the study	
☒	☐	ChIP-seq
☒	☐	Flow cytometry
☒	☐	MRI-based neuroimaging

Human research participants

Policy information about studies involving human research participants

Population characteristics	See above.
Recruitment	Children with ASD were recruitedthrough community resources, including local chapters of the Autism Society ofAmerica, local public and private schools, as well as clinics at KennedyKrieger Institute (KKI) as were children with ADHD, in particular the Center for Autism and RelatedDisorders (CARD). Prior to initial entry, all children recruited will undergo an initial telephone screening lasting 15 to 20 minutes with a parent to determine their general suitability for inclusion or exclusion. Children of all race and gender were included, as long as they were between 8-12 yrs old.
Ethics oversight	Studies in which these data were collected were/are under the approval of the Kennedy Krieger Institute and the Johns

Ethics oversight	Hopkins School of Medicine Institutional Review Boards.

Note that full information on the approval of the study protocol must also be provided in the manuscript.
